# A randomized controlled trial to evaluate the effectiveness of a staff training program to implement consumer directed care on resident quality of life in residential aged care

**DOI:** 10.1186/s12877-018-0966-1

**Published:** 2018-11-23

**Authors:** Marita P. McCabe, Elizabeth Beattie, Gery Karantzas, David Mellor, Kerrie Sanders, Lucy Busija, Belinda Goodenough, Michelle Bennett, Kathryn von Treuer, Jessica Byers

**Affiliations:** 1 School of Health Sciences, Swinburne University of Technology, H95 PO BOX 218, Hawthorn, VIC 3122 Australia; 2 The Dementia Centre for Research Collaboration, Queensland University of Technology, Brisbane, Australia; 3 School of Psychology, Deakin University, Geelong, Australia; 4 Department of Medicine, University of Melbourne and Western Health, Sunshine Hospital, Melbourne, Australia; 5 Monash University, Melbourne, Australia; 6 Dementia Training Australia, University of Wollongong, Melbourne, NSW Australia; 7 School of Allied Health, Australian Catholic University, Sydney, Australia; 8 Cairnmillar Institute, Melbourne, Australia

**Keywords:** Consumer directed care, Staff training, Resident quality of life, Residential aged care, Resident choice and control

## Abstract

**Background:**

Residential Aged Care Facilities (RACFs) are moving towards a Consumer Directed Care (CDC) model of care. There are limited examples of CDC in ageing research, and no evaluation of a comprehensive CDC intervention in residential care was located. This study will implement and evaluate a staff training program, Resident at the Center of Care (RCC), designed to facilitate and drive CDC in residential care.

**Methods:**

The study will adopt a cluster randomized controlled design with 39 facilities randomly allocated to one of three conditions: delivery of the RCC program plus additional organizational support, delivery of the program without additional support, and care as usual. A total of 834 staff (22 in each facility, half senior, half general staff) as well as 744 residents (20 in each facility) will be recruited to participate in the study. The RCC program comprises five sessions spread over nine weeks: Session 1 clarifies CDC principles; Sessions 2 to 5 focus on skills to build and maintain working relationships with residents, as well as identifying organizational barriers and facilitators regarding the implementation of CDC. The primary outcome measure is resident quality of life. Secondary outcome measures are resident measures of choice and control, the working relationship between resident and staff; staff reports of transformational leadership, job satisfaction, intention to quit, experience of CDC, work role stress, organizational climate, and organizational readiness for change. All measures will be completed at four time points: pre-intervention, 3-months, 6-months, and 12-month follow-up. Primary analyses will be conducted on an intention to treat basis. Outcomes for the three conditions will be compared with multilevel linear regression modelling.

**Discussion:**

The RCC program is designed to improve the knowledge and skills of staff and encourage transformational leadership and organizational change that supports implementation of CDC. The overarching goal is to improve the quality of life and care of older people living in residential care.

**Trial registration:**

ACTRN12618000779279; Registered 9 May 2018 with the Australian and New Zealand Clinical Trials Registry (ANZCTR; http://www.anzctr.org.au/).

## Background

The residential aged care system in Australia and internationally is striving towards a Consumer Directed Care (CDC) model, an approach to resident care that emphasizes consumer choice and control. This model of care places the residents at the center of care, such that the residents have choice and control over their care and activities. CDC is designed to improve the residents’ quality of life (QoL) by supporting them in directing the design and delivery of the services they receive [1]. Introducing CDC will require Residential Aged Care Facilities (RACFs) to move away from a traditional approach to care, which is typically driven by the routines and efficiencies of the organization [2]. RACFs will need to adapt this prevailing mindset and logistics in order to support the dignity, autonomy, and independence of residents [3] and more comprehensively meet individual resident care preferences. There are currently considerable gaps in our knowledge of how to achieve and sustain this model of care. Research on implementation and efficacy of CDC is limited, with most published CDC studies focusing on its delivery in community aged care [4, 5]. While person-centered approaches have been evaluated in the aged care context with some positive outcomes [6, 7], no specific CDC-based staff training programs have been evaluated in RACFs. Our project will address this gap by providing an innovative approach to RACF staff training so that they can effectively implement CDC in residential care.

Challenges associated with implementing a resident-directed approach in aged care include the lack of staff empowerment to handle the shift towards CDC philosophy, the need for substantial job restructuring, resistance to change, and the need for strong leadership to support practice change [8, 9]. Implementing a CDC approach therefore requires training the care staff and facility management in CDC strategies (for example, implementing resident choice and control regarding activities and daily routines) as well as focusing on change management and leadership strategies. In particular, a transformational style of leadership is central to translating the knowledge and skills relevant to CDC into practice. Transformational leaders are focused on change [10], and are able to bring about awareness and acceptance of mission and purpose within the organization that results in a wide variety of positive outcomes [11]. As such, they are more likely to engage and generate positive attitudes about the required organizational change among care staff when compared to non-transformational leaders [8, 12].

The organizational climate is critical to translating the CDC strategies into practice. A fundamental change in how a RACF functions under CDC is the focus on each resident’s participation in the planning and delivery of the services they receive, as opposed to the efficiency of care delivery. Thus the focus is on reorganizing the activities of the facility to address the expressed needs of the residents, rather than being focused on tasks identified by facility staff. Aspects of organizational change and staff relationships, such as role clarity and innovation [13], work pressures, commitment and trust, will be essential to this shift. Our previous work with 255 aged care workers across 21 RACFs demonstrated that, in addition to transformational leadership, work pressure and innovation were predictive of aged care employees’ perceptions of organizational readiness for change [14]. Work pressure may be predictive of readiness for change if workers perceive that the change will alleviate pressure [14], while innovation indicates that the organization encourages change and creativity [15]. It is important to examine the role of all of the factors as they relate to the implementation of CDC.

Beyond organizational factors, working relationships between RACF care staff and residents are likely to be central to the success of CDC and improvement of resident QoL. Positive interactions between staff and residents can build trust and give residents a sense of respect and acknowledgment, which, in turn, can be the foundation for the ongoing collaboration needed to support residents’ involvement in their care [16]. Additionally, meaningful relationships, as determined from both the resident and staff perspectives, are expected to improve both resident quality of life [17] and staff job satisfaction [12, 18].

Our Resident at the Center of Care (RCC) training program consists of five sessions and is directed at managers and general staff. It has been developed to address the critical factors outlined below (transformational leadership, organizational climate, and the working relationship between staff and residents) in order to drive real and sustainable change in implementing and embedding CDC in residential aged care, and so improve resident QoL. Education (training) alone is necessary but insufficient for driving practice change [19]. Further, large scale reforms like CDC are disruptive to existing practice and likely to be viewed ambivalently by staff [20]. It is therefore important to embed knowledge translation strategies into the RCC program to enable change.

### Hypotheses and expected outcomes

It is hypothesized that, relative to care as usual, the RCC Program will improve residents’ QoL (Primary Outcome Measure – QoL at 6-months follow-up); their perceptions of their level of choice and control and the working relationship with staff (Secondary Outcome Measures). It is also hypothesized that the RCC Program will lead to organizational change; improve staff perceptions of their use of transformational leadership, job satisfactions, working relationship with residents, experience of CDC; reduce their job stress and intention to quit (Secondary Outcome Measures). Supplementing the RCC Program with extra support to assist staff to address barriers to CDC implementation and so implement change is hypothesized to lead to greater benefits than the same program without this support. These changes are predicted to occur, relative to baseline at 3-months, 6-months, and 12-months follow-up.

## Methods/design

### Study design

A cluster randomized controlled trial design will be employed in this study, with 39 facilities randomly allocated to one of three conditions (13 facilities in each). Each of the 13 aged care organizations will provide three separate facilities to take part in the study, which will then be randomly allocated to one of the three conditions. Allocations of facilities will be completed by the biostatistician not involved in data collection using random numbers generated at random.org.

**Fig. 1:**
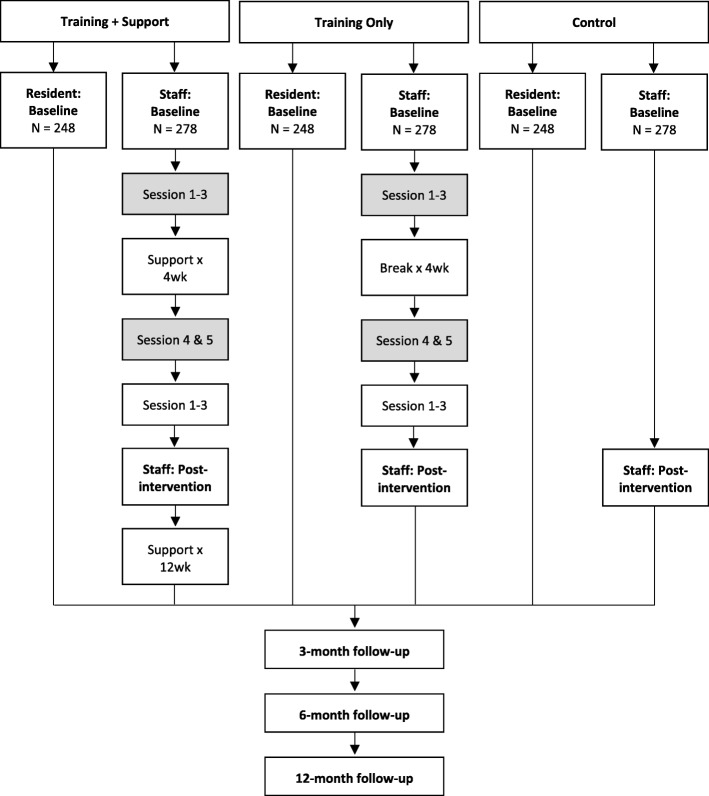
Flow chart of staff recruitment, baseline, roll out of the program, post-intervention and follow-up

The first condition will involve the delivery of the five-session RCC Program plus additional organizational support. This organizational support is designed to assist staff to implement the CDC model of care within their facility. It will be provided one day a week for four weeks following Session 3 and a further one day a week for 12 weeks following Session 5. This training is above and beyond what is provided within each session of the training program, and is designed to assist staff in applying their learnings from the program (in line with the facility’s CDC implementation plan) and assist the staff to work through challenges as they arise. Sessions 4 and 5 will occur immediately after a four-week gap (see Fig. 1). The second condition will be a second intervention group, where staff will be provided with the RCC Program without the additional organizational support. The third condition will be a ‘care as usual’ control group. Staff will be provided with paid release time to attend the program. Data from the residents will be collected by a research assistant who is blind to the condition to which the facility is allocated.

A 0.5 FTE study champion appointment will be made by each organization. This study champion will be recruited from the commencement of the study, with a brief to work with staff and the research team to assist in the roll out of the strategies identified as a result of the training program. This study champion will assist in the recruitment of staff and residents for the study as well as assist staff to embed the strategies from the program into routine practice. The study champion will be an important link between the staff in the two intervention facilities and the research team implementing the protocol. In addition to the organizational support provided for Condition 1, the study champion will assist staff to overcome barriers to CDC implementation throughout the 15 months of the study period. The overall study will run from January 2018 through to December 2020. The first round of staff and resident recruitment and data collection commenced in May 2018 and it is anticipated the final round of data collection will be completed by July 2020. The roll-out of the training program will run from June 2018 to July 2019. The second half of 2020 will be dedicated to data analysis and interpretation, and writing of publications.

Informed by the results of our earlier NHMRC-funded RCT (APP1042156) our study is designed to detect a moderately small difference *d* ≥ 0.35 between the RCC plus organizational support and control Condition in QoL of residents at 6-months follow up (primary outcome) and in staff outcomes post-intervention (secondary outcomes). We selected 6-months follow up as our primary endpoint for residents’ QoL because we expect a gradual and continuing change in this outcome as a result of changing practice. Allowing for 80% power, 5% Type I error rate (2-tailed), intra-class correlation of 0.02 for the within-facility clustering, and 25% (residents) and 33% (staff) attrition over time, we will need to randomize at least 744 residents (248 per Condition) and 834 staff (278 per Condition). Based on our previous work in the residential aged care setting, we expect an average recruitment of 20 residents and 22 staff per site. Hence, 39 aged care facilities will need to be enrolled into the study (13 for each arm of the RCT). Data analysis is discussed later in the Methods/Design section.

### Consent and ethics

This project has been approved by the university Human Research Ethics Committee. All participating aged care staff and aged care residents will be provided with a plain language statement and will be asked to provide written informed consent to participate. For staff, the consent will be to participate in the staff training and/or questionnaire completion. For residents, the consent will be sought to access their records at the facility to obtain health-related information and collect self-reported quality of life assessments. If residents are unable to provide consent (e.g. due to impaired cognitive function), consent will be sought from their next of kin. There will be no reimbursement for residents or staff participating in the study.

### Participants

A total of 834 staff (22 in each facility) will participate in the study. In order to be eligible to participate in the study, staff members will have to be a full-time, part-time, or casual permanent employee at the facility, and be either a member of the senior or general staff. Approximately equal numbers of senior (e.g. registered nurse) and general (e.g. personal care attendant) staff across a range of roles will be recruited at each RACF. Staff employed on a contractual basis from an external organization will not be permitted to take part in the study.

A total of 744 residents (20 in each facility) will also be recruited to participate in the study. Residents will be eligible to participate in the study if they: (a) have lived in the facility for longer than 3 months; (b) can effectively communicate in English; (c) are not considered to have a severe cognitive impairment, based on their most recent Psychogeriatric Assessment Scale (PAS) score [21]; (d) are aged 65 years and older; and (e) are physically able to participate. After potential participants have been identified by the facilities, they will be approached by a member of the research team with an invitation to take part in the study.

### Intervention program

The translation strategy utilized in the RCC used the Dementia Training 4-stage ‘Awareness-Agreement-Adoption-Adherence’ approach to designing continuing education for health professionals [19]. The first session is designed to engage the senior staff only, orienting them to the program scope and goals, and their critical role as leaders during the training program and beyond. It aims to clarify the key CDC principles and how they relate to residential aged care. The key factors that may pose a challenge or help facilitate the successful implementation and sustainability of CDC will be explored, along with the key organizational factors (i.e. staff autonomy and recognition; workplace fairness and innovation; trust; support and cohesion). Senior staff will also be introduced to tools such as the Resident Care Form, which will be used to foster the collaborative working relationship between care staff and residents. The Resident Care Form will be used to elicit resident choices on a range of areas of care (e.g., meals, time for showers, activities, etc.). This session also discusses the importance of leadership in facilitating change, specifically transformational leadership. Senior staff will then be encouraged to adopt a transformational leadership style when guiding the participation of other staff members in the remainder of the program.

Sessions 2 to 5 will involve both senior and general staff. Sessions 2 and 3 will review the philosophy and approach of CDC, and explore staff members’ prior knowledge, ideas and experiences of CDC. The importance of building and maintaining working relationships with residents will be emphasized. The Resident Care Form will be introduced to general staff as a tool to support residents’ choice and control over their care decisions. In these sessions, senior and general staff will also discuss the factors that may hinder or promote successful implementation and sustainability of CDC.

There will be a 4-week break from training between Sessions 3 and 4, in which care staff will complete the Resident Care Forms with residents and practice implementing any changes requested by residents in their activities. During this time, facilities that have been allocated to Condition 1 (the RCC Program plus support) will also receive weekly support from a member of the research team to assist them to address any barriers and foster enablers to the implementation of CDC. The study champion will also assist in this process. Following the training break, staff members will be asked to review their experience with the Resident Care Form, and draw on learnings from previous sessions to develop a CDC implementation plan during Sessions 4 and 5. Following the end of the training program, the staff at sites allocated to Condition 1 (the RCC Program plus support) will receive a further 12 weeks of support to implement their CDC plans for one day a week.

### Evaluation of the training program

#### Evaluation measures for aged care residents

The following measures will be completed by the residents at baseline and 3 months, 6 months, and 12 months follow-up. A research assistant who is blind to the condition will individually sit with each resident and work through the measures listed below.

#### Quality of life

Residents’ self-reported QoL will be assessed using the Quality of Life – Alzheimer’s Disease (QoL-AD) Aged Care Adaptation questionnaire [22]. Residents will rate 15 different aspects of their life (e.g. physical health, mood, memory, functional ability, interpersonal relationships and engagement in meaningful activities) on a 4-point scale from ‘Poor’ to ‘Excellent’. Higher scores indicate higher quality of life (possible range: 0 to 60). Previous research on this scale has demonstrated high reliability (α = .92) [22].

#### Residents’ choice and control

The Duncan Choice Index [23] will be used to assess residents’ perception on the amount of choice available to them in leisure and self-care activities. The scale consists of 28 quantitative items that will be categorized under headings, including Eating, Grooming, and Leisure. Responses are recorded on a 3-point scale: ‘Never’, ‘Sometimes, and ‘Always’, with higher scores indicating a higher frequency of choice opportunities (possible range: 28–84). Each category will include an open-ended question that inquires whether the resident would like to have more choice in that particular area. The scale was reported to have a Cronbach’s alpha of .84 [23].

#### Working relationship

Residents’ perceptions of the extent to which their care staff support their autonomy will be assessed using Perceived Autonomy Support: The Health Care Climate Questionnaire [24]. This scale contains 15 items, which will be adapted to suit the residential aged care setting. Responses are recorded on a 3-point scale, with higher scores indicating a more positive perception of support to the resident’s autonomy (possible range: 15 to 45). Previous research on this scale has demonstrated high reliability (α = .92) [24].

#### Evaluation measures for aged care staff

The following measures will be completed by care staff at all residential facilities at baseline, post-intervention and 3 months, 6 months, and 12 months follow-up.

#### Transformational leadership

The Multifactor Leadership Questionnaire (MLQ) – Leader version by Bass and Avolio [25] will be used to assess the senior staff’s perception of their own leadership style. The scale consists of 45 items in 9 subscales that can be condensed into 4 subscales. Responses are recorded on a 5-point scale, with higher scores on each subscale indicating the extent to which a staff member employs a particular leadership style. The scale has demonstrated good reliability, with the alpha for the subscales ranging from .61 to .91 [25].

#### Job satisfaction

An 8-item questionnaire drawn from Tate, Whately, and Clugston [26] will be used to measure the costs and rewards associated with an individual’s job. Staff will respond on a 5-point Likert scale, with higher scores indicating greater job satisfaction (possible range: 8 to 40). The scale has been reported to have good reliability (α = .85) [26].

#### Intention to quit

Staff members will respond to two questions from Tate et al. [26] to rate their intention and likelihood to leave their current job. Responses are provided on a 5-point scale, with higher scores indicating a greater intention to quit (possible range: 2 to 10). Previous research has demonstrated acceptable reliability for this scale (α = .61) [26].

#### Working relationship

Staff members’ perception of their relationships with residents will be assessed using the Scale to Assess Therapeutic Relationships in Community Health Care, Clinician version (STAR-C) [27]. The scale contains 12 items, which will be adapted to better suit the residential aged care setting and sample population. Staff members will respond using a 5-point Likert Scale, with a higher total score indicating a more positive working relationship (possible range: 0 to 44). The scale has been reported to have acceptable test-retest reliability (*r* = .92) [27].

#### Experience of CDC

The Individualized Care Scale by Chappell and colleagues [28] will be used to measure staff members’ perception on the extent to which principles of CDC are already practiced in their facility. The scale consists of four sub-scales. *Knowing the Person* (α = .77) measures staff members’ perception of how well they know the resident they are caring for as a person. It contains 13 items, and responses are recorded using a 4-point scale. Higher scores indicate that carers believe they know the residents well (possible range: 13–52). *Resident Autonomy* (α = .80) contains 8 items which assess staff members’ perception of how much control and choice the residents have in their everyday environment and activities that are related to their care. This scale uses a 5-point scale, with higher scores indicating a belief that the facility in which the staff member is employed supports the residents’ autonomy (possible range: 8–40). *Staff-to-resident Communication* (α = .64) refers to the extent to which care staff use communication that is not task-focused and communicate with residents about the residents’ care needs and preferences. Responses to the 7 items in this subscale are recorded on a 4-point scale, with higher scores describing a lower frequency of task-focused forms of communication between staff and residents (possible range: 7–28). *Staff-to-staff Communication* (α = .76) measures how actively staff members communicate with one another about residents’ needs and preferences and about facility procedures and practices. Responses to the 11 items in these subscales are recorded on a 4-point scale, with higher scores indicating more frequent exchange of information among staff members regarding resident care (possible range: 11–44).

Additionally, staff opinions about potential advantages and disadvantages of the CDC approach will be assessed using 6 open ended questions. The questions will only be completed by staff in Condition 1 and 2, following Session 3 of the training program (when staff are familiar with CDC) and at 6-month follow-up. These questions will be used to determine if their understanding and perception of CDC has altered following the implementation of CDC strategies in the facility.

#### Work role stress

The Strain in Dementia Care Scale [29] will be used to assess the extent to which care staff members experience stress while caring for residents. The scale consists of 27 items which ask staff to indicate how often they experience a stressful situation, thought, or feeling in their role as a carer, and how much stress it causes them when it occurs. Responses are recorded on a 4-point scale, with higher scores indicating a higher frequency of the occurrence and higher stress (possible range: 27–108). The scale has been reported to have satisfactory internal consistency (α = .89) [29].

#### Person-centered care

The Person-centered Care Assessment Tool (P-CAT) will be used to measure the extent to which the care within a facility is experienced by staff as being person-centered [30]. The scale contains 13 items. Responses are recorded on a 5-point scale, with higher scores indicating the staff member’s perception that the care approach within their facility adheres to person-centered care approaches (possible range: 13–65). Previous research using the scale has reported good reliability (α = .84) [30].

#### Staff perceptions of organizational climate

This construct will be measured using the Organizational Climate Questionnaire (OCQ) [15]. The scale consists of eight subscales that constitute organizational climate: trust, autonomy, fairness, innovation, pressure, cohesion, support, and recognition. Each of these subscales contains 5 questions and has good reliability (α > .80) [15]. Responses are recorded on a 5-point scale, with higher scores indicating a more positive perception of the organizational climate (possible range: 40 to 200).

#### Readiness for organizational change

This four-factor 25-item scale measures current perceptions of the organization [31]. Responses are recorded on a 5-point scale. Coefficient alphas for the four factors were .94 for *Appropriateness*, .66 for *Personally Beneficial*, .87 for *Management Support* and .82 for *Change Efficacy* [31]. This scale will only be completed after Session 3 of the training program and at 6-month follow-up to determine if the organization’s readiness to change has altered due to the implementation of the program and practice in CDC strategies.

### Statistical analysis

Primary analyses will be on an intention to treat basis, with supplementary ‘per protocol’ analyses (at least 75% of staff enrolled in the study completing at least 75% of training sessions). To account for the within-facility clustering and repeated assessments of staff and residents, hypothesized main effects for outcomes will be tested using a multilevel linear (or generalized linear, where appropriate) regression modelling. For each outcome, a separate three-level regression model will be specified, with repeated measurements as level 1, individuals as level 2, and facility as level 3. The regression models will include Condition allocation, assessment time point, and Condition by time interaction as predictors. Facilities and individuals will be modelled as random effects with remaining variables modelled as fixed effects. Missing data will be handled with conditional maximum likelihood estimation. The impact of possible non-random attrition will be explored with simulation analyses.

## Discussion

Implementing and sustaining the CDC model of care in RACFs requires training the facility’s care staff and management to move away from a task-focused approach to prioritizing residents’ choice and control. The project described in this article will provide and evaluate a training program for staff designed to address the critical aspects of CDC: enhancement of transformational leadership, organizational factors, and the working relationship between staff and residents. It is expected that the training program will result in organizational change, improved levels of residents’ choice, control and quality of life, as well as staff members’ perception of their work environment. Overall, it is expected that this training program will facilitate a smooth transition into CDC. All residential aged care facilities participating in the RCC program (Condition 1 and 2) are expected to demonstrate the above improvements relative to the control condition (Condition 3). The RCC program plus support (Condition 1) is expected to show greater adoption/adherence to the implementation of CDC, and so the greatest improvements in the above variables in comparison to Condition 2 and 3.

This project aims to transform practice in the aged care sector by improving the knowledge and skills of RACFs’ staff regarding CDC, thus improving the quality of life and care of residents. It will also equip staff to address organizational barriers jeopardizing the implementation of CDC, and empower staff to lead in a style that enhances the implementation of CDC. Moreover, the RCC training program will serve as a platform for the future national and international roll out of training programs for staff to ensure the uptake of CDC.

## Abbreviations

CDC: Consumer directed care
MLQ: Multifactor leadership questionnaire
OCQ: Organizational climate questionnaire
PAS: Psychogeriatric assessment scale
P-CAT: Person-centered care assessment tool
QoL: Quality of life
QoL-AD: Quality of life – Alzheimer’s disease
RACFs: Residential aged care facilities
RCC: Resident at the center of care
